# Perspective on the New American Heart Association/American College of Cardiology Guidelines for the Management of Adults with Congenital Heart Disease

**DOI:** 10.19102/icrm.2018.091209

**Published:** 2018-12-15

**Authors:** George F. Van Hare

**Affiliations:** ^1^Department of Pediatrics, Washington University School of Medicine, St. Louis, MO, USA

**Keywords:** Congenital heart disease, guidelines, pediatrics

Recently, new guidelines for the management of adults with congenital heart disease (CHD) were released by the American Heart Association and the American College of Cardiology.^1^ These guidelines represent an update to the previously published 2008 guidelines^2^ and incorporate quite a bit of new information. The document represents several years of incredibly hard work on the part of the first author and leader of the document, Karen Stout, as well as of her coauthors. I am proud to be one of these coauthors. As the study group points out, the prevalence of adult CHD is increasing, primarily due to the incredible successes that have occurred in pediatric cardiology and pediatric cardiothoracic surgery that have allowed patients with even the most severe congenital heart defects to reach adulthood. Pediatric cardiologists and cardiac surgeons alike can be justly proud of what they have accomplished in the care of infants with critical CHD. Most of us feel very attached to our teenaged and young adult CHD survivors, and we are now placing a high priority on promoting an effective transition for them to adult congenital care.

The management of these patients truly represents a new specialty in the field of cardiology, as these individuals often exhibit novel clinical problems that are certainly not limited to the cardiovascular system. Often, science advances when clinicians are confronted by new patient populations with novel medical problems and so, we are at a particularly exciting juncture in the field of cardiology. In addition to having the opportunity to ensure that these patients live as long and as productive lives as possible, there are tremendous scientific and academic opportunities available to cardiologists working in this field.

As electrophysiologists, we have always known that the repair of these defects, even when completely successful from a hemodynamic standpoint, often leads to downstream electrical issues. Indeed, for many patients who reach adulthood with repaired CHD, their most significant and troubling issues are in the area of postoperative arrhythmias.

The Pediatric and Congenital Electrophysiology Society (PACES), in partnership with the Heart Rhythm Society (HRS), recently published a comprehensive expert consensus statement on the management of arrhythmias in adults with repaired CHD.^3^ This extensive document covered all aspects of arrhythmias in all forms of CHD in adults and had more than 500 references. Thus, the more recent AHA/ACC document did not attempt to redo that work but rather instead sought to focus on some of the most important issues facing adults with CHD with respect to arrhythmias.

First and foremost, comprehensive adult CHD programs need electrophysiology expertise. Just as the field of adult CHD is distinct from and requires different expertise versus both the fields of pediatric cardiology and adult cardiology, so too is adult congenital electrophysiology developing as its own separate field. The expertise in adult congenital electrophysiology may come from either pediatric or adult electrophysiology programs, or ideally from both in partnership. While an understanding of the congenital and surgical anatomy of these patients is important in addressing their arrhythmias, particularly in the ablation suite and for device implantation, the effects of aging are equally if not more important and the significance of atrial fibrillation in this patient population cannot be overstated. With respect to available facilities, the electrophysiology laboratory should be equipped with three-dimensional mapping and other advanced mapping technologies as well as the necessary professional expertise.

Many might find it reassuring that the role of electrocardiography was confirmed in this document. While it may seem obvious, obtaining electrocardiograms at the time of routine clinic visits is quite important, as occult arrhythmias are fairly common in this patient population—including, for example, slow intra-atrial reentry tachycardia (IART)—and, as they may not always be symptomatic, detecting them during routine follow-up is important. Similarly, sinus node dysfunction and junctional rhythm are often seen and have important implications. Most cardiologists are aware of the importance of the QRS duration as a risk factor for ventricular tachycardia and sudden death in patients following the repair of tetralogy of Fallot as an easily obtained index of right ventricular dilation. Finally, it is important not to forget Wolff-Parkinson-White syndrome, which is often seen in patients with Ebstein’s anomaly and can occasionally be acquired following specific forms of heart surgery.

Similarly, ambulatory electrocardiography (Holter monitoring) as well as the deployment of various approaches to event monitoring are important in this patient population, as atrial and ventricular arrhythmias can develop with time and with changes in patients’ hemodynamic status.

Two forms of repaired CHD were highlighted with respect to the risk of arrhythmias. First, patients with dextro-transposition of the great arteries who have previously undergone an atrial switch procedure (ie, Senning or Mustard) are known to have a high incidence of IART, and the document highlights the importance of understanding a patient’s congenital anatomy in order to perform effective catheter ablation for this arrhythmia, which usually originates from structures on the pulmonary venous side of the interatrial baffle. Second, there is a large focus on tetralogy of Fallot and related anatomies, including the problem of risk stratification for the possibility of sudden cardiac death in these patients. The importance of cardiac magnetic resonance imaging is highlighted, as is the utility of programmed ventricular stimulation to seek inducible sustained monomorphic ventricular tachycardia. In fact, the latter is perhaps one of the only remaining indications for diagnostic ventricular programmed stimulation! The document comments on the use of implantable cardioverter-defibrillator (ICD) placement as primary prevention in tetralogy of Fallot patients with multiple risk factors for sudden cardiac death, which is considered a class IIa indication. Finally, the document discusses the importance of protecting the right ventricle as a way of preventing the development of the substrate for ventricular tachycardia and sudden death through appropriate timing of pulmonary valve replacement, either surgically or by transcatheter techniques.

The spectrum of adults with CHDs is broad and encompasses patients with well-repaired defects with little need for frequent cardiology follow-up at one end to those patients who are most severely affected, such as postoperative Fontan patients who require very close follow-up in specialized adult congenital centers, at the other. Helpfully, the document categorizes the various forms of repaired CHD into a number of categories based on severity and provides helpful tables indicating both the ideal frequency of adult CHD clinic follow-up as well as the specific testing methods, including arrhythmia monitoring and other procedures, to be included as part of routine follow-up. While much of the information in this document will primarily be helpful to specialists in the adult CHD field, there is plenty here for pediatric cardiologists, adult cardiologists, and electrophysiologists who are confronted from time to time with adults with CHD.

## References

[1] Stout KK, Daniels CJ, Aboulhosn JA (2018). 2018 AHA/ACC guideline for the management of adults with congenital heart disease: executive summary: a report of the American College of Cardiology/American Heart Association Task Force on Clinical Practice Guidelines.. J Am Coll Cardiol..

[2] Warnes CA, Williams RG, Bashore TM (2008). ACC/AHA 2008 guidelines for the management of adults with congenital heart disease: a report of the American College of Cardiology/American Heart Association Task Force on Practice Guidelines (Writing Committee to Develop Guidelines on the Management of Adults With Congenital Heart Disease). Developed in Collaboration With the American Society of Echocardiography, Heart Rhythm Society, International Society for Adult Congenital Heart Disease, Society for Cardiovascular Angiography and Interventions, and Society of Thoracic Surgeons.. J Am Coll Cardiol..

[3] Khairy P, Van Hare GF, Balaji S (2014). PACES/HRS expert consensus statement on the recognition and management of arrhythmias in adult congenital heart disease: developed in partnership between the Pediatric and Congenital Electrophysiology Society (PACES) and the Heart Rhythm Society (HRS). Endorsed by the governing bodies of PACES, HRS, the American College of Cardiology (ACC), the American Heart Association (AHA), the European Heart Rhythm Association (EHRA), the Canadian Heart Rhythm Society (CHRS), and the International Society for Adult Congenital Heart Disease (ISACHD).. Heart Rhythm..

